# Effect of Processing Intensity on Immunologically Active Bovine Milk Serum Proteins

**DOI:** 10.3390/nu9090963

**Published:** 2017-08-31

**Authors:** Tabea Brick, Markus Ege, Sjef Boeren, Andreas Böck, Erika von Mutius, Jacques Vervoort, Kasper Hettinga

**Affiliations:** 1 Dr. von Hauner Children’s Hospital, Ludwig Maximilians University Munich, Lindwurm Str. 4, 80337 Munich, Germany; tabea.brick@med.uni-muenchen.de (T.B.); A.Boeck@med.uni-muenchen.de (A.B.); Erika.Von.Mutius@med.uni-muenchen.de (E.v.M.); 2 Comprehensive Pneumology Centre Munich (CPC-M), Member of the German Center of Lung Reseach (DZL), 80337 Munich, Germany; 3 Laboratory of Biochemistry, Wageningen University, 6708 WE Wageningen, The Netherlands; sjef.boeren@wur.nl (S.B.); jacques.vervoort@wur.nl (J.V.); 4 Helmholtz Zentrum München—German Research Center for Environmental Health, Institute for Asthma and Allergy Prevention, Ingolstädter Landstr. 1, 85764 Neuherberg, Germany; 5 Dairy Science and Technology, Food Quality and Design Group, Wageningen University, 6708 PB Wageningen, The Netherlands; kasper.hettinga@wur.nl

**Keywords:** proteomics, heat stability, milk serum proteins, immune-active proteins

## Abstract

Consumption of raw cow’s milk instead of industrially processed milk has been reported to protect children from developing asthma, allergies, and respiratory infections. Several heat-sensitive milk serum proteins have been implied in this effect though unbiased assessment of milk proteins in general is missing. The aim of this study was to compare the native milk serum proteome between raw cow’s milk and various industrially applied processing methods, i.e., homogenization, fat separation, pasteurization, ultra-heat treatment (UHT), treatment for extended shelf-life (ESL), and conventional boiling. Each processing method was applied to the same three pools of raw milk. Levels of detectable proteins were quantified by liquid chromatography/tandem mass spectrometry following filter aided sample preparation. In total, 364 milk serum proteins were identified. The 140 proteins detectable in 66% of all samples were entered in a hierarchical cluster analysis. The resulting proteomics pattern separated mainly as high (boiling, UHT, ESL) versus no/low heat treatment (raw, skimmed, pasteurized). Comparing these two groups revealed 23 individual proteins significantly reduced by heating, e.g., lactoferrin (log2-fold change = −0.37, *p* = 0.004), lactoperoxidase (log2-fold change = −0.33, *p* = 0.001), and lactadherin (log2-fold change = −0.22, *p* = 0.020). The abundance of these heat sensitive proteins found in higher quantity in native cow’s milk compared to heat treated milk, renders them potential candidates for protection from asthma, allergies, and respiratory infections.

## 1. Introduction

Consuming raw milk has been associated with a reduction in risk of childhood asthma and atopy [1,2] as well as respiratory infections [3]. However, consumption of raw milk poses significant risks, due to potential presence of pathogens in raw milk [3]. As an alternative to raw milk, specific milk ingredients for supplementing heat treated milk have become the focus of recent research, and a wide range of components have been hypothesized to be related to the allergy and asthma protective potential of raw milk versus commercially available milk [4].

After industrial processing, cow’s milk considerably differs from raw milk in several aspects, with fat content and heat-treatment being the most obvious. Although the effects of fat content and heat treatment on reduction of asthma partially overlap, both factors exert strong independent effects [2]. The effect of fat content was mainly attributed to the levels of ω-3 polyunsaturated fatty acids [2]. Similarly, heat treatment reduced the levels of milk serum proteins such as β-lactoglobulin and α-lactalbumin, which in turn were found to be inversely related to asthma risk in children of the GABRIELA study with statistical significance [1].

Although it remains open whether these proteins actually reduce the asthma risk themselves, these findings suggest an allergy preventive potential by heat-sensitive proteins in general. Generally whey proteins are susceptible to heat treatment [5,6], particularly immunoactive proteins such as lactoferrin or lactadherin [7]. Heating of heat-labile proteins results in denaturation and aggregation processes [8] and thereby leads to a loss of biological functionality (e.g., lactoferrin [9]). Denatured and aggregated proteins can be extracted from the milk with a combination of pH reduction and ultracentrifugation [10], after which remaining levels of non-aggregated milk serum proteins can be determined [7]. Besides denaturation and aggregation, heating may also lead to chemical modifications, especially the Maillard reaction [11]. During industrial milk processing, relatively short heating times are applied, thus we expect relatively low levels of such chemical modifications, although especially for UHT processing a certain level of chemical modifications has previously been observed [12]. The aim of this study was to assess the native protein profile of bovine milk serum after different industrially applied processing steps with varying heating intensity for the identification of potential asthma- and allergy-protective candidate proteins.

## 2. Materials and Methods

### 2.1. Milk Samples

The milk samples used for this analysis were derived from three different farms located in Southern Germany. The origins and characteristics of these three milk batches are shown in Table 1. Each milk batch was processed on three consecutive days in a pilot plant. From milk collection to the last processing step, milk samples were stored at 1 °C. After processing, the milk samples were stored at −20 °C until proteomics analysis. The milk types resulting from the various processing procedures are listed in Table 2. Industrial milk processing was not done with technical replicates because these procedures are laborious, expensive, and time-consuming and there were biological replicates represented by the three milk batches from the respective farms. In total, eight milk samples from each of the three milk batches were assessed for proteomics. The same 24 milk samples were previously used to assess the effect of different processing methods on microRNA (miRNA) levels [13].

The subsequent proteomics analyses of the 24 samples including sample preparation and mass spectrometry were performed without technical replicates since technical reproducibility proved to be high in previous experiments [14] and most of the variation was expected to come from the three separate batches of milk.

### 2.2. Removal of Fat and Denatured Protein

All samples were centrifuged at 1500× *g* for 10 min at 10 °C (with a rotor 25.15, Avanti Centrifuge J-26 XP, Beckman Coulter, Miami, FL, USA). After centrifugation, all skimmed milk samples were acidified by drop-wise addition of 1 M HCl under stirring, until a pH of 4.6 was reached. The samples were then kept at 4 °C for 30 min to equilibrate. When needed, pH was adjusted before the final pH reading. This pH adjustment was done to separate the denatured serum proteins from the native serum proteins during ultracentrifugation, as previously described [7,10]. The acidified skim milk was transferred to ultracentrifuge tubes followed by ultracentrifugation at 100,000× *g* for 90 min at 30 °C (Beckman L-60, rotor 70 Ti). After ultracentrifugation, samples were separated into three phases. The top layer was remaining milk fat, the middle layer was milk serum, and the bottom layer (pellet) was casein with denatured proteins. Milk serum was used for filter aided sample preparation (FASP) as described below.

### 2.3. Filter Aided Sample Preparation (FASP)

FASP method was carried out according to Wisniewski et al., 2009 [15], with adaptations according to Zhang et al., 2016 [7]. Milk serum samples (20 μL) were diluted in SDT-lysis buffer (4% SDS with 0.1 M dithiotreitol and 100 mM Tris/HCl pH 8.0) to get a 1 μg/μL protein solution. Samples were then incubated for 10 min at 95 °C. They were centrifuged at 21,540× *g* for 10 min after being cooled down to room temperature. Of each sample 20 μL were directly added to the middle of 180 μL 0.05 M iodoacetamide (IAA) in 8 M urea with 100 mM Tris/HCl pH 8.0 (called UT) in a low binding Eppendorf tube and incubated for 10 min while mildly shaking at room temperature. The entire volume of the sample (200 μL) was transferred to a Pall 3K omega filter (10–20 kDa cutoff, OD003C34; Pall, Washington, NY, USA) and centrifuged at 20,000× *g* for 30 min. Another three centrifugations at 20,000× *g* for 30 min were carried out after adding three times 100 μL UT. Afterwards 110 μL 0.05 M NH_4_HCO_3_ (ABC) in water was added to the filter unit and centrifuged at 20,000× *g* for 30 min. Then, the filter was transferred to a new low-binding Eppendorf tube. On the filter, 100 μL ABC containing 0.5 μg trypsin was added and centrifuged at 20,000× *g* for 30 min after incubation overnight. Finally, the filter was removed and 5 μL 10% trifluoroacetic acid (TFA) was added to adjust the pH of the sample to around 2. These samples were ready for analysis by liquid chromatography/tandem mass spectrometry (LC-MS/MS).

### 2.4. LC-MS/MS Analysis

A volume of 18 μL of the trypsin digested milk fractions was injected in a 0.10 × 30 mm Magic C18AQ 200A 5 µm beads (Bruker Nederland B.V., Leiderdorp, The Netherlands) pre-concentration column (prepared in house) at a maximum pressure of 270 bar. Peptides were eluted from the pre-concentration column onto a 0.10 × 200 mm Magic C18AQ 200A 3 µm beads analytical column with an acetonitrile gradient at a flow of 0.5 μL/min, using gradient elution from 8 to 33% acetonitrile in water with 0.5 *v*/*v* % acetic acid in 50 min. The column was washed using an increase in the percentage of acetonitrile to 80% (with 20% water and 0.5 *v*/*v* % acetic acid in the acetonitrile and the water) in 3 min. Between the pre-concentration and analytical columns, an electrospray potential of 3.5 kV was applied directly to the eluent via a stainless steel needle fitted into the waste line of a P777 Upchurch microcross. Full scan positive mode FTMS spectra were measured between *m*/*z* 380 and 1400 on a LTQ-Orbitrap XL (Thermo electron, San Jose, CA, USA) in the Orbitrap at high resolution (60,000). IT and FT AGC targets were set to 10,000 and 500,000, respectively, or maximum ion times of 100 µs (IT) and 500 ms (FT) were used. Collision-induced dissociation (CID) fragmented MS/MS scans (isolation width 2 *m*/*z*, 30% normalized collision energy, activation Q 0.25 and activation time 15 ms) of the four most abundant 2+ and 3+ charged peaks in the FTMS scan were recorded in data dependent mode in the linear trap (MS/MS threshold = 5.000, 45 s exclusion duration for the selected *m*/*z* ±25 ppm).

### 2.5. Data Analysis

Each run with all MS/MS spectra obtained was analysed with Maxquant 1.3.0.5 with Andromeda search engine [16]. Carbamidomethylation of cysteines was set as a fixed modification (enzyme = trypsin, maximally 2 missed cleavages, peptide tolerance for the first search 20 ppm, fragment ions tolerance 0.5 amu). Oxidation of methionine, N-terminal acetylation and de-amidation of asparagine or glutamine were set as variable modification for both identification and quantification. The bovine reference database for peptides and protein searches was downloaded as fasta file from Uniprot with reverse sequences generated by Maxquant (fasta file downloaded from Uniprot 2013 [17]). A set of 31 protein sequences of common contaminants was used as well, which included Trypsin (P00760, bovine), Trypsin (P00761, porcine), Keratin K22E (P35908, human), Keratin K1C9 (P35527, human), Keratin K2C1 (P04264, human), and Keratin K1C1 (P35527, human). A maximum of two missed cleavages were allowed and a mass deviation of 0.5 Da was set as limit for MS/MS peaks and maximally 6 ppm deviation on the peptide *m*/*z* during the main search. The false discovery rate (FDR) was set to 1% on both peptide and protein levels. The length of peptides was set to at least seven amino acids. Finally, proteins were displayed based on minimally 2 distinct peptides of which at least one unique and at least one unmodified. Match between runs was used with a time window of 10 min. Both unmodified and modified peptides were used for quantification. Only unique or razor peptides were used for quantification. Minimum ratio count for label-free quantification (LFQ) was set as 2.

The quantification of the full proteome is based on the extracted ion current and is taking the whole three-dimensional isotope pattern into account, using peak volumes of all measured isotopes for quantification [16]. At least two quantitation events were required for a quantifiable protein. MaxQuant was used with the Intensity based absolute quantification (IBAQ) algorithms for quantification [18]. The IBAQ algorithm estimates the absolute amount of a protein as the sum of the intensities of all peptides (based on peak volumes), divided by the number of tryptic peptides that can theoretically be generated. Proteins had to have at least three valid IBAQ intensities in the individual samples for counting of the number of identified proteins.

The function of the identified proteins was checked in the UniprotKB database released February 2014 [17].

### 2.6. Statistical Analysis

Statistical analysis was performed with R 3.3.2 software [19]. The average number of measurable proteins in raw milk was calculated and related to the respective numbers of milks after different processing methods.

Proteins with ≤33% non-detects were included in further analysis. Non-detects of these proteins were either simply replaced by zero or imputed by simple imputation. For the imputation firstly the mean and standard deviation of each protein was estimated including the non-detected values as censored observations by a linear Tobit model to determine protein specific distributions. Subsequently, non-detects were replaced by random samples from the lower tail of the respective distribution, i.e., below the protein specific detection limit as defined by the minimum of the measured protein levels. The quality of imputation was examined via Wilcoxon tests, comparing median protein levels of the imputed data against the raw data. For subsequent analyses, the imputed data were used.

Hierarchical clustering of milk samples was based on Pearson’s correlation of the specific protein profiles following imputation.

For assessment of the effect of heating, milks were categorized in two groups by temperatures above and below 80 °C [20]; high heated milk samples, defined as UHT, ESL and boiled milk and no-/low heat treated milks, represented by pasteurized, skimmed and raw milk samples (Table 2).

A logistic regression model adjusted for the milk origin (Traunstein, Freising, Starnberg) was used to calculate the differences in high vs. low heat treated milks. The log2 fold-changes of the protein levels in low versus high heat treated milks were calculated to rank the proteins according to their heat sensitivity, and plotted against the corresponding negative decadic logarithm of the *p*-values in a volcano plot. Resulting *p*-values were adjusted for the false discovery rate according to Benjamini–Hochberg, and a corrected *p*-value < 0.05 was considered statistically significant.

## 3. Results

A total of 364 milk serum proteins were identified and quantified in at least one of the 24 milk samples, of which 44 could be quantified in all 24 samples. Subsequent analyses were based on the 169 proteins found in at least three different milk samples; 130 of those proteins were detected in all three raw milk samples and further 28 proteins in two raw milk samples. The average LFQ levels of proteins in the raw milk samples did not differ significantly between the three farms (*p* = 0.49), thereby ruling out major differences in original milk batches.

**Figure 1:**
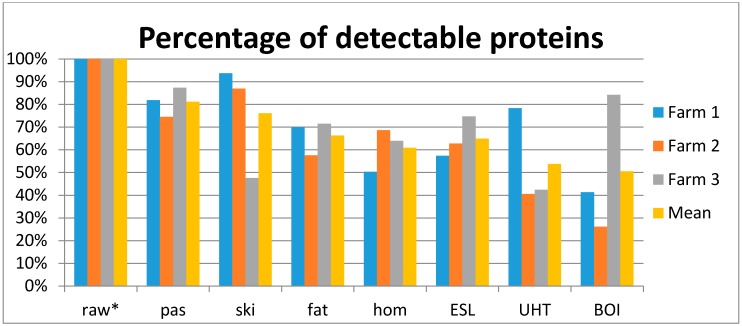
Proportion of number of detectable proteins in milk samples (each sample per farm individually and averaged over the three different samples) after different processing compared to raw cow’s milk. * No. of detectable native proteins in raw milk is the reference, i.e., 151 distinct proteins were detected in the three raw milk samples on average.

Figure 1 shows a substantial loss of detectable proteins after the various processing procedures.

**Figure 2:**
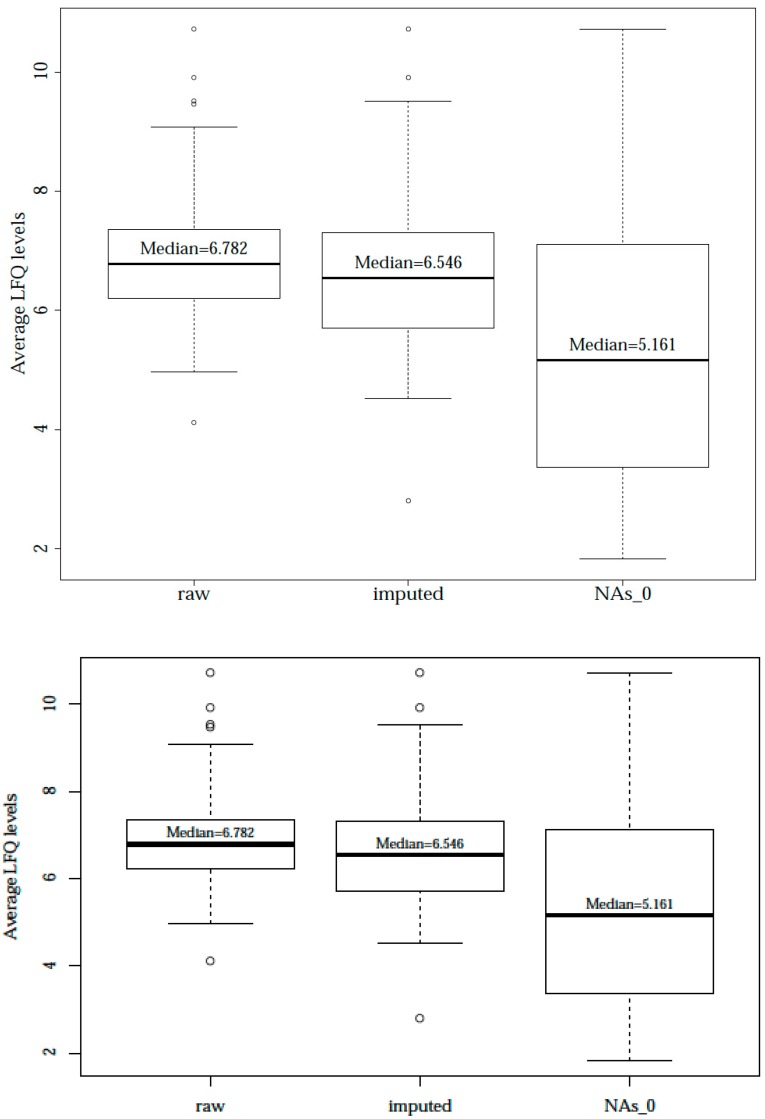
Boxplots of average protein LFQ levels after different NA replacement. Comparison of mean LFQ protein values in different data (raw, imputed, and NAs replaced by 0). Replacement by 0 differed significantly from the raw data (*p* < 0.0001).

For further statistical analysis, proteins with >33% non-detects were excluded. Non-detects in the remaining proteins (*n* = 140) were either replaced by imputed values below detection limit or simply by zeros. Figure 2 shows the superiority of the imputation method in contrast to the simple replacement of missing values by zero. The median of the individual protein LFQ levels averaged over all 24 samples is solely slightly reduced after imputation compared to the raw data set (median value was calculated after exclusion of missing values). In contrast, replacement of non-detects by zero resulted in a clear distortion of the distribution and was not considered for further analysis.

**Figure 3:**
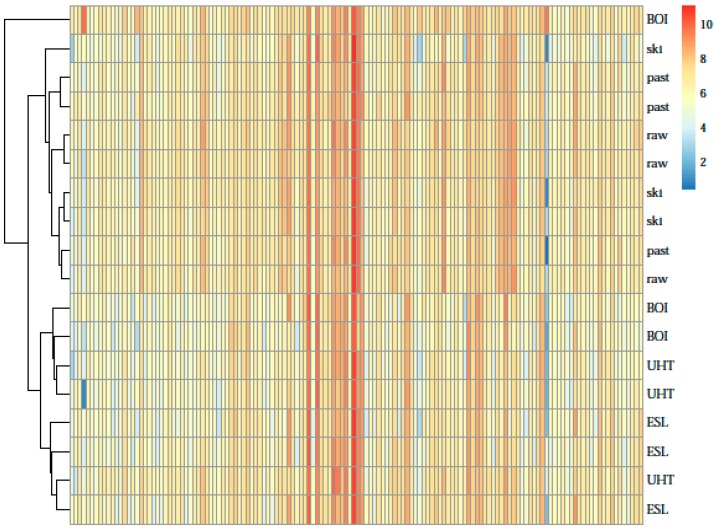
Heat map for protein levels and milk types. Rows reflect individual samples, whereas individual proteins are given in columns. Their LFQ values are represented by different colors according to the color code from low (blue) to high (red) expression.

Similar protein patterns resulted from similar heating temperatures of the milk samples as demonstrated by hierarchical clustering of the specific protein profiles (Figure 3): raw, skimmed and pasteurized milk samples formed one cluster, whereas UHT, ESL, and boiled milk samples formed another cluster with the exception of one boiled milk sample, which differed substantially from both main clusters. Under the assumption that this milk was partially overcooked, it was excluded from subsequent analyses.

**Figure 4:**
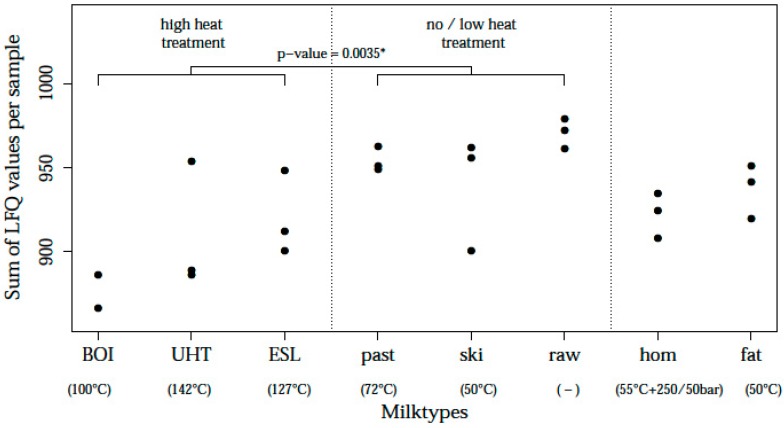
Total protein contents (sum of LFQ values per sample) in differently processed milks. * *p*-value derived from a logistic regression with adjustment for milk batch.

Comparison of milks in the high heat versus the low heat treated group revealed a significant reduction of the total protein LFQ levels in high heated milks compared to no/low heat treated milks, as shown in Figure 4. Boiled milk showed the lowest protein levels; other heat treated milks contained total protein LFQ levels ranging between boiled and raw milk and were inversely related to heating temperatures (Figure 4).

**Figure 5:**
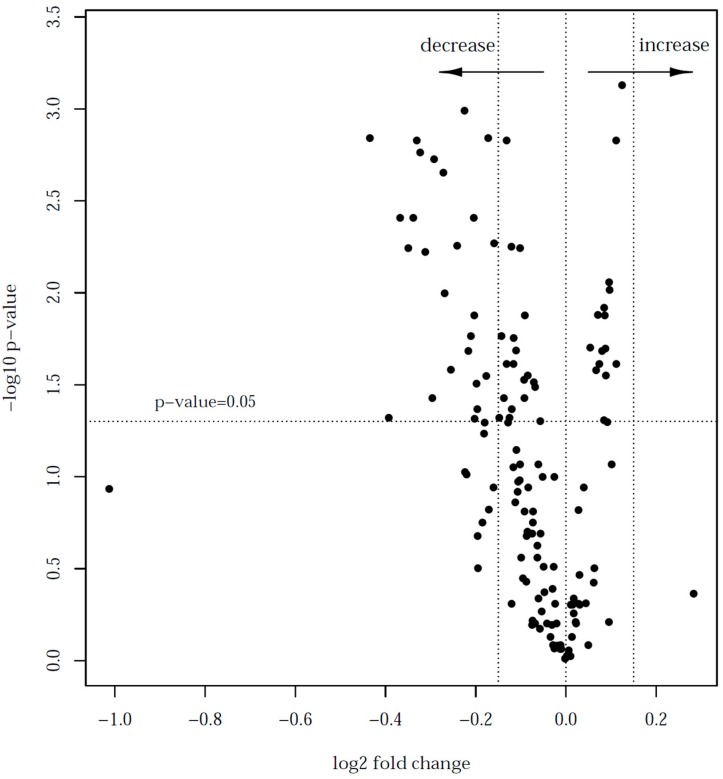
Volcano plot for the comparison of proteins in low- versus high-heat treated milk samples. The log two-fold change of protein expression between low- and high-heat treated milk samples is plotted against the corresponding *p*-values from a *t*-test given as negative decadic logarithm. A negative log two-fold change indicates a decrease in LFQ levels.

When focusing on individual proteins, a significant reduction in quantity of at least 10% was found in 23 proteins after high heat treatment compared to low heat treatment (Figure 5).

Ten of these proteins were related to immune functions (Table 3).

## 4. Discussion

Heat treatment of milk led to a considerable decrease in number of detectable proteins and their levels of quantification with a clear relationship to the applied heat load. The most intensive treatment, i.e., boiling, reduced the number of proteins that could be detected by about 50% compared to raw milk, with the other heating types ranging in between. The various processing methods led to specific proteomic patterns covering 140 individual proteins as demonstrated by a cluster analysis. Of these, 23 distinct proteins were found to be substantially diminished in high heat treated milks. The majority of these heat-sensitive proteins were related to immune functions.

Typically, people in Westernized countries consume industrially processed milk and, increasingly, milk types with an extended shelf life. In addition, UHT milk with its very long storage duration of three months or more is nowadays very popular. Traditionally, commercially available milk had been pasteurized, i.e., heated at 72 °C for 20 s to inactivate potential hazardous microorganisms with only small gain in shelf life.

Despite the potential risk of life-threatening infections, a minority of people still consume raw cow’s milk, which has repeatedly been reported to protect against asthma, allergies, and respiratory infections in childhood [1,3,21,22]. The wide consumption of cow’s milk thus renders it an attractive strategy for prevention if the risk of infections were to be overcome. An option might be the isolation and purification of the protective milk ingredients, and various studies have focused on the impact of industrial processing on the potentially beneficial molecules. At the same time, reducing heat load of commercially available dairy products may already lead to an increase in the availability of potentially immunoactive proteins.

Of the industrially applied processing steps, predominantly fat separation for adjusting milk fat levels, and homogenization for preventing fat creaming, affect the milk lipid fraction. However, homogenization also leads to a massive increase in fat globule surface, which will be covered by milk proteins, leading to a reduction of milk proteins in serum.

Waser at al., 2007 [23] found an asthma and wheeze protective effect of milk fat containing products such as full cream milk and butter. In addition, Brick et al., 2016 [2] implied the higher fat content and more precisely the higher content of anti-inflammatory omega-3 fatty acids in raw milk in the asthma protective effect of full cream milk obtained directly from a farm. Despite mild heating to 55 °C, high pressure treatment of milk (250 bar) used during the homogenization process has been found to profoundly rearrange protein quantity and structure [22,24,25]. In addition, in the present study homogenization reduced total protein LFQ levels and specific protein detectability markedly.

Another major processing step is heating for destroying hazardous microorganisms and increasing shelf life. Thermo-labile milk components such as miRNAs [13] and proteins may thus be involved in the protective effect of raw milk. Particularly protein functionality, solubility and quantity are all affected by intensity of heat treatment [7,8,18,26]. Previously specific miRNA species were identified as possible contributors to the asthma-protective effect of farm milk [11]. This notion is not in conflict with our current findings; rather both molecule classes might add to the effect or might even interact.

Loss et al., 2011 [1] found inverse associations of asthma with higher levels of several milk whey proteins, i.e., bovine serum albumin, alpha-lactalbumin and beta-lactoglobulin. However, it remains unclear whether these specific proteins confer the effect themselves or whether they are proxies of heat labile proteins in general. Therefore, we quantified heat-induced alterations of the entire milk proteome by a comprehensive, standardized, and unbiased approach, i.e., without preselection of proteins.

First, we observed a considerable decrease of detectable proteins after heat treatment in a dose-dependent manner. Boiled cow’s milk contained the lowest number of detectable proteins, which is explained by the high heat load applied. The lower temperature of boiling compared to ESL or UHT is more than compensated by the much longer duration of the heating (Table 2). In addition, the long heating time of boiling may also lead to more extensive chemical modification compared to industrial processes [11], further reducing protein levels in these samples.

For further investigation in the impact of heating on the protein quantity and heat sensitivity, milk samples were categorized into high heat and no/low heat treated milk groups according to the clusters presented in the heat map (Figure 3). This dichotomization was in line with findings on the first marginal transition of bovine whey proteins at about 81 °C [20]. Actually, the difference between high and no/low heat treated samples was more than 25 °C with pasteurization not exceeding 72 °C and high heat treatment starting with 100 °C.

Figure 1 and Figure 4 describe some variance within milk types between the three farms, e.g., one of the UHT samples had a higher percentage of detectable proteins and a higher summed LFQ value than the respective other two UHT samples. Nevertheless, the heat map (Figure 3) still groups all the high-heated samples together. Even though this UHT sample contains a higher overall protein intensity, and a higher number of identified proteins, the proteome profile still reflects a high heated sample. This might be due to a similar pattern in decrease of individual, heat sensitive proteins. The exact underlying mechanisms for these individual variations however cannot be explained in this study. Further investigations on a larger scale are needed to better understand the variability in proteome profile after heat processing.

The sum over all proteins, and more specifically the levels of 23 individual proteins were substantially lower in high heat treated samples as expected by previous work from Zhang et al., 2016 [7]. Interestingly, most of these 23 particularly heat-sensitive proteins were related to immune functions (Table 3), and several proteins have already been mentioned in the context of asthma and allergies. Under the assumption that some proteins withstand the acidity of the stomach milieu, they may be resorbed in the gut and exert physiologic functions. At least this has been suggested for e.g., lactoferrin (LTF) [27], protease inhibitors [28] and IgG [29].

Among the most promising candidates was lactoferrin, which is known to stimulate the immune system by counteracting pathogenic invaders and injuries and preventing harmful overreactions of the immune system [30,31].

Lactoperoxidase is a peroxidase enzyme secreted from the mammary gland that operates as a natural antibacterial agent [32]. Asthmatic patients who were treated with lactoperoxidase aerosol showed lower disease activity and reduced damaging effects of hydrogen peroxide (H_2_O_2_), which is mainly generated by neutrophils and eosinophils in asthma and contributes to airway damages and inflammation [33].

Xanthine dehydrogenase/oxidase (XOR) might contribute to the formation of NO in the intestinal lumen and thereby exert antimicrobial properties [34]. In our study we were unable to differentiate the rather similar variants, dehydrogenase and oxidase, as the only difference is an intramolecular change of two cysteines in the disulfide bond, whereas the amino acid chain, analyzed with the LC-MS/MS analysis, is identical.

In addition, a number of acute phase proteins such as fibrinogen, prothrombin, complement C3 and C9 were found to be highly heat-sensitive. How they may be involved in the anti-inflammatory effects ascribed to raw milk remains unclear, although the complement pathway, and specifically C3, has been implied in the development of allergy and asthma [35,36,37].

Plakophilin-3 acts protective in both local and systemic inflammatory diseases [38] and inter-alpha-trypsin inhibitor has anti-inflammatory, anti-scarring and anti-angiogenic properties [39]. Protease inhibitors, including several inter alpha-trypsin inhibitors, have been found to be upregulated in the breast milk of allergic mothers and have been related to the pathogenesis of allergy and asthma [40,41].

Lactadherin expression is found to be markedly reduced in asthmatic patients compared to healthy subjects, and suppresses airway smooth muscle hypercontractility [42].

Polymeric immunoglobulin receptor may influence eosinophilic inflammation by binding secretory immunoglobulins [43]. In addition, secretory components, which are part of the polymeric immunoglobulin receptor that can be cleaved off, have shown individual effects in mucosal immunity [44].

Ultimately, the discovery of the CD14 molecule, a receptor of bacterial endotoxin, is interesting as gene–environment interactions of raw milk consumption and polymorphisms associated with this gene have been discussed controversially for childhood onset asthma [45,46]. Similar to the human CD14 molecule, its bovine counterpart might transmit signals elicited by endotoxin, and thereby have an effect on the development or prevention of allergy and asthma.

Despite the plausible involvement of several proteins in the beneficial health effects we have to acknowledge that we cannot provide a direct link to disease status in this study. However, the palette of immune-active milk components detected in the present study can be seen as an extension to the findings by Loss et al., 2011 [1], which explicitly linked protein levels to disease. In addition, this study only shows a decrease in native proteins, due to either denaturation or heat-induced chemical modification, without direct evidence for a loss-of-function. However, heating of milk has been shown to reduce biological activity of milk, including antibacterial capacity [6] and previous studies showed a loss-of-function of milk immune proteins upon denaturation (e.g., Paulson, 1993 [47]; Marin et al., 2003 [48]). However, future studies are needed to investigate in the biological function of milk’s immunoactive proteins after applying heat treatments.

Another limitation of this analysis is the omission of the milk fat globule membrane (MFGM) fraction [49]; their relatively low abundance in cow’s milk, however, precludes a major contribution to the effects by the entirety of immunoactive proteins present in milk. Our analyses were made after one freezing cycle; resulting alterations, however, seem to be very limited [7,50].

## 5. Conclusions

Taken together, we have performed a comprehensive search for proteins most likely to be affected by industrial processing methods. Their higher abundance in native cow’s milk as compared to industrially processed milks renders them potential candidates for protection from asthma, allergies, and respiratory infections. However, in this study, we solely analyzed protein patterns of differently processed milks, thus associations of found potential protein candidates with disease status have to be investigated in population based studies.

## Figures and Tables

**Table 1:** Sources of raw milk.

Sample Origin	Farms (in Bavaria)		
	Traunstein	Freising	Starnberg
No. of cows	13	60	30
Time point of milking for pooled samples	Morning and evening	Morning and evening	Morning and evening
No. of detectable milk serum proteins in raw milk samples	143	153	158

**Table 2:** Processing details of the milk samples.

Code	Milk Fraction	Processing Conditions	Day of Processing *	Grouping of Milk Types **
RAW	Native raw milk	-	Wednesday	No-low heat
PAS	Pasteurized	72 °C for 20 s	Wednesday	No-low heat
		Total processing time *** 60 s		
SKI	Skim milk	Separation at 50 °C	Tuesday	No-low heat
FAT	Fat fraction/cream	Separation at 50 °C	Tuesday	-
HOM	Homogenized milk	Preheating to 55 °C, 2-stage homogenization at 250/50 bar	Tuesday	-
ESL	Extended shelf life milk	Preheating at 95 °C for 20 s, direct steam injection at 127 °C for 5 s	Monday	High heat
		Total processing time *** 60 s		
UHT	Ultra-high heat treated	Preheating at 93 °C for 23 s, direct steam injection at 142 °C for 5 s	Monday	High heat
		Total processing time *** 85 s		
BOI	Boiled milk	Preheating at >80 °C for >300 s, boiling at 100 °C for 30 s	Tuesday	High heat
		Total processing time *** 2000 s		

* Milk samples were collected on a Monday and stored at 1 °C until they were processed. Processing occurred on the same day or the two subsequent days. After processing samples were frozen to −20 °C and stored until analysis. ** For further analysis of heat treatment on milk proteins, grouping of milk types according to the heat treatment was conducted; homogenized milk was excluded due to additional treatment with pressure; cream was excluded because it contains only the milk fat fraction. *** Total processing time includes heating and cooling stage.

**Table 3:** Significantly differing proteins between high and no/low heat treated milk-types with a change of ≥10%.

Protein Code	Number of Peptides	*p*-Value *	Log2 Fold Change (95% CI)	Protein Name	Protein Function
P80457	67	0.001	−0.44 (−0.56; −0.31)	Xanthine dehydrogenase/oxidase	immunity
P24627	71	0.004	−0.37 (−0.51; −0.22)	Lactoferrin	immunity
G3X6N3	57	0.006	−0.35 (−0.50; −0.20)	Serotransferrin	transport
F1MR22	42	0.004	−0.34 (−0.47; −0.21)	Polymeric immunoglobulin receptor	immunity
P80025	37	0.001	−0.33 (−0.43; −0.23)	Lactoperoxidase	immunity
G3N1R1	4	0.002	−0.32 (−0.44; −0.21)	Uncharacterized protein	unknown
F1MGU7	7	0.04	−0.30 (−0.52; −0.07)	Fibrinogen gamma-B chain	Blood coagulation
G3X7A5	80	0.002	−0.29 (−0.41; −0.18)	Complement C3	immunity
F1MZ96	10	0.002	−0.27 (−0.36; −0.18)	Uncharacterized protein	unknown
F1MX50	4	0.01	−0.27 (−0.40; −0.13)	Uncharacterized protein	cell
F1MM32	8	0.026	−0.26 (−0.43; −0.08)	Sulfhydryl oxidase	enzyme
P81265	42	0.006	−0.24 (−0.35; −0.14)	Polymeric immunoglobulin receptor	immunity
F1N076	12	0.001	−0.23 (−0.30; −0.15)	Ceruloplasmin	cell
F1MXX6	26	0.02	−0.22 (−0.35; −0.08)	Lactadherin	cell
Q08DQ0	6	0.017	−0.21 (−0.34; −0.08)	Plakophilin-3	cell
P07589	6	0.004	−0.20 (−0.30; −0.11)	Fibronectin	immunity
A6QNL0	6	0.01	−0.20 (−0.32; −0.09)	Monocyte differentiation antigen CD 14	immunity
P10152	11	0.048	−0.20 (−0.37; −0.04)	Angiogenin-1 (ribonuclease 5)	cell
F1MMD7	5	0.031	−0.20 (−0.34; −0.06)	Inter-alpha-trypsin inhibitor heavy chain H4	Protease inhibitor
Q3MHN2	6	0.043	−0.20 (−0.35; −0.04)	Complement component C9	immunity
P00735	7	0.028	−0.18 (−0.30; −0.05)	Prothrombin	immunity
F1MCF8	9	0.001	−0.17 (−0.22; −0.12)	Uncharacterized protein	immunity
P17690	9	0.005	−0.16 (−0.23; −0.09)	Beta-2-glycoprotein 1	Blood coagulation

* *p*-values are adjusted for multiple testing.

## Supplementary Materials

The supplementary file is available online at www.mdpi.com/2072-6643/9/9/963/s1.
