# Supplementary material for: Effect of Processing Intensity on Immunologically Active Bovine Milk Serum Proteins
Source: Nutrients. 2017 Aug 31;9(9):963. doi: 10.3390/nu9090963 (PMC5622723; doi:10.3390/nu9090963)
Supplement: Supplementary file 1 [file nutrients-09-00963-s001.pdf]

nutrients

MDPI

Supplemental Table S1: Differentiation of Table 3.

Protei n	p-Value	Difference logscale	Full protein name	Protein function	farm
A6QNL0	0.081671945	**-0.23802192**	**Monocyte differentiation antigen CD14**	immunity	**1**
F1MCF8	0.034750355	**-0.17906498**	**Uncharacterized protein**	immunity	**1**
F1MGU7	0.256887543	**-0.29739410**	**Fibrinogen gamma-B chain**	blood coagulation	**1**
F1MM32	0.114634543	**-0.37410274**	**Sulfhydryl oxidase**	enzyme	**1**
F1MMD7	0.490982158	**-0.13948175**	**Inter-alpha-trypsin inhibitor heavy chain H4**	protease inhibitor	**1**
F1MR22	0.084272485	**-0.40955884**	**Uncharacterized protein**	immunity	**1**
F1MUT3	0.138322440	**-0.23642798**	**Xanthine dehydrogenase/oxidase**	enzyme	**1**
F1MX50	0.155685884	**-0.23493682**	**Uncharacterized protein**	cell	**1**
F1MXX6	0.196097111	**-0.21122983**	**Lactadherin**	cell	**1**
F1MZ96	0.028505851	**-0.29591851**	**Uncharacterized protein**	unknown	**1**
F1N076	0.050156461	**-0.23059976**	**Uncharacterized protein**	cell	**1**
G3N1R1	0.081971360	**-0.35570269**	**Uncharacterized protein**	unknown	**1**
G3X6N3	0.093323121	**-0.41136014**	**Serotransferrin**	transport	**1**
G3X7A5	0.074969423	**-0.32985784**	**Complement C3**	immunity	**1**
P00735	0.246247618	**-0.20028194**	**Prothrombin**	immunity	**1**
P07589	0.249664417	**-0.13446460**	**Fibronectin**	immunity	**1**
P10152	0.262017558	**-0.24632455**	**Angiogenin-1**	cell	**1**
P14923	0.442842564	**-0.23557883**	**Junction plakoglobin**	contaminant	**1**
P17690	0.067252125	**-0.19795256**	**Beta-2-glycoprotein 1**	blood	**1**

				coagulation	
P24627	0.618396262	**-0.05641995**	**Lactotransferrin**	immunity	**1**
P80025	0.056354987	**-0.31864315**	**Lactoperoxidase**	immunity	**1**
P80457	0.024660244	**-0.48824513**	**Xanthine dehydrogenase/oxidase**	enzyme	**1**
P81265	0.030342765	**-0.23337220**	**Polymeric immunoglobulin receptor**	immunity	**1**
Q08DQ0	0.408093065	**-0.18463537**	**Plakophilin-3**	cell	**1**
Q3MHN2	0.161391410	**-0.13610006**	**Complement component C9**	immunity	**1**
A6QNL0	0.069013882	**-0.23518111**	**Monocyte differentiation antigen CD14**	immunity	**2**
F1MCF8	0.015784629	**-0.20428409**	**Uncharacterized protein**	immunity	**2**
F1MGU7	0.083850004	**-0.30868066**	**Fibrinogen gamma-B chain**	blood coagulation	**2**
F1MM32	0.006709007	**-0.39112003**	**Sulfhydryl oxidase**	enzyme	**2**
F1MMD7	0.105977817	**-0.28763775**	**Inter-alpha-trypsin inhibitor heavy chain H4**	protease inhibitor	**2**
F1MR22	0.051009420	**-0.43844310**	**Uncharacterized protein**	immunity	**2**
F1MUT3	0.008728533	**-0.16801522**	**Xanthine dehydrogenase/oxidase**	enzyme	**2**
F1MX50	0.039875202	**-0.36328489**	**Uncharacterized protein**	cell	**2**
F1MXX6	0.067107745	**-0.28572580**	**Lactadherin**	cell	**2**
F1MZ96	0.033714785	**-0.29583862**	**Uncharacterized protein**	unknown	**2**
F1N076	0.014556345	**-0.23929233**	**Uncharacterized protein**	cell	**2**
G3N1R1	0.079212935	**-0.24260691**	**Uncharacterized protein**	unknown	**2**
G3X6N3	0.058801881	**-0.36820290**	**Serotransferrin**	transport	**2**
G3X7A5	0.025457293	**-0.33343274**	**Complement C3**	immunity	**2**
P00735	0.189244691	**-0.18823088**	**Prothrombin**	immunity	**2**
P07589	0.001882570	**-0.16339849**	**Fibronectin**	immunity	**2**

P10152	0.265167328	**-0.16016051**	**Angiogenin-1**	cell	**2**
P14923	0.066201298	**-0.26988851**	**Junction plakoglobin**	contaminant	**2**
P17690	0.034045350	**-0.17825876**	**Beta-2-glycoprotein 1**	blood coagulation	**2**
P24627	0.606428473	**-0.10691213**	**Lactotransferrin**	immunity	**2**
P80025	0.015727966	**-0.40803462**	**Lactoperoxidase**	immunity	**2**
P80457	0.017164248	**-0.45127591**	**Xanthine dehydrogenase/oxidase**	enzyme	**2**
P81265	0.076082396	**-0.31250532**	**Polymeric immunoglobulin receptor**	immunity	**2**
Q08DQ0	0.568689620	**-0.07368748**	**Plakophilin-3**	cell	**2**
Q3MHN2	0.038221029	**-0.32713635**	**Complement component C9**	immunity	**2**
A6QNL0	0.808810787	**-0.04043559**	**Monocyte differentiation antigen CD14**	immunity	**3**
F1MCF8	0.102303854	**-0.11513041**	**Uncharacterized protein**	immunity	**3**
F1MGU7	0.530105296	**-0.26148465**	**Fibrinogen gamma-B chain**	blood coagulation	**3**
F1MM32	0.366806380	**-0.30728700**	**Sulfhydryl oxidase**	enzyme	**3**
F1MMD7	0.706563764	**-0.03510671**	**Inter-alpha-trypsin inhibitor heavy chain H4**	protease inhibitor	**3**
F1MR22	0.290188981	**-0.28563547**	**Uncharacterized protein**	immunity	**3**
F1MUT3	0.053002049	**-0.56981654**	**Xanthine dehydrogenase/oxidase**	enzyme	**3**
F1MX50	0.324407131	**-0.19072733**	**Uncharacterized protein**	cell	**3**
F1MXX6	0.382993501	**-0.11720980**	**Lactadherin**	cell	**3**
F1MZ96	0.317901367	**-0.19368184**	**Uncharacterized protein**	unknown	**3**
F1N076	0.082822215	**-0.19684531**	**Uncharacterized protein**	cell	**3**
G3N1R1	0.237873253	**-0.28592347**	**Uncharacterized protein**	unknown	**3**
G3X6N3	0.238346830	**-0.23144136**	**Serotransferrin**	transport	**3**

G3X7A5	0.203309875	**-0.18251452**	**Complement C3**	immunity	**3**
P00735	0.435981155	**-0.07472569**	**Prothrombin**	immunity	**3**
P07589	0.074917993	**-0.25812729**	**Fibronectin**	immunity	**3**
P10152	0.329023124	**-0.22825551**	**Angiogenin-1**	cell	**3**
P14923	0.717283262	**-0.17966716**	**Junction plakoglobin**	contaminant	**3**
P17690	0.142082287	**-0.07824901**	**Beta-2-glycoprotein 1**	blood coagulation	**3**
P24627	0.891718223	**-0.02615023**	**Lactotransferrin**	immunity	**3**
P80025	0.146780262	**-0.23888622**	**Lactoperoxidase**	immunity	**3**
P80457	0.226280758	**-0.32739478**	**Xanthine dehydrogenase/oxidase**	enzyme	**3**
P81265	0.147299243	**-0.15649395**	**Polymeric immunoglobulin receptor**	immunity	**3**
Q08DQ0	0.482109041	**-0.20910686**	**Plakophilin-3**	cell	**3**
Q3MHN2	0.465662798	**-0.19406143**	**Complement component C9**	immunity	**3**
